# Adrenocortical Oncocytic Carcinoma and Papillary Thyroid Carcinoma Incidentally Detected in an Asymptomatic Patient by F-18 FDG PET/CT 

**DOI:** 10.22038/aojnmb.2018.10845

**Published:** 2018

**Authors:** Batool Al Balooshi, Shabna Miyanath, Amr Elhennawy, Yaser Saeedi, Syed Hammad Tirmazy, Muhammed Muhasin, Bhavna Ray, Mouza Al Sharhan, Hassan Hotait, Yamina Houcinat, Tasnim Keloth

**Affiliations:** 1 Dubai nuclear Medicine and Molecular maging Center, Dubai Hospital, Dubai Health Authority, Dubai, UAE; 2 Department of Urology, Dubai Hospital, Dubai Health Authority, Dubai, UAE; 3 Department of Oncology, Dubai Hospital, Dubai Health Authority, Dubai, UAE; 4 Department of Pathology and Genetics, Dubai Hospital, Dubai Health Authority, Dubai, UAE

**Keywords:** Adrenal mass, Adrenal oncocytoma, Adrenocortical carcinoma, F-18 FDG, Thyroid carcinoma

## Abstract

F-18 FDG is the most widely used tracer in molecular imaging and it is applied for many purposes mainly in malignant diseases. Incidental finding are common in FDG-PET/CT imaging and includes benign and malignant lesions. Among the rare tumors‎, adrenal oncocytomas are uncommon findings and incidental findings of thyroid malignancies are not rare. Oncocytoma is a rare adrenocortical tumor and majority of bulky adrenal tumors are benign with uncertain incident of malignancy. In this study, we are reporting a 37-year-old man with two incidental malignancies detected by FDG-PET-CT. He has no symptoms has no blood and hormonal abnormalities.

The scan demonstrated intense heterogeneous FDG uptake within the bulky oval shaped lesion in the left adrenal gland. Accordingly, open adrenalectomy was performed and diagnosis of adrenocortical carcinoma oncocytic type was established. Furthermore, a focal FDG uptake was identified in the right thyroid lobe and histopathology findings were consistent with well-differentiated papillary thyroid cancer. FDG plays a great role in identifying primary rare lesions and also detection of incidental findings at unexpected sites.

## Introduction

Oncocytoma is a rare adrenal tumor usually diagnosed incidentally and maybe found in other organs too (1). Increasing use of F-18 FDG imaging resulted in incidental finding of asymptomatic cancers (2-4). Although FDG is not a specific tracer for cancer, however using appropriate criteria may play a vital role in the interpretation of incidental F-18 FDG PET/CT findings. We report for the first time a patient with two rare association; adrenal nonfunctioning oncocytic carcinoma and thyroid papillary carcinoma detected using F-18 FDG PET/CT.

## Case report

**Figure 1.**
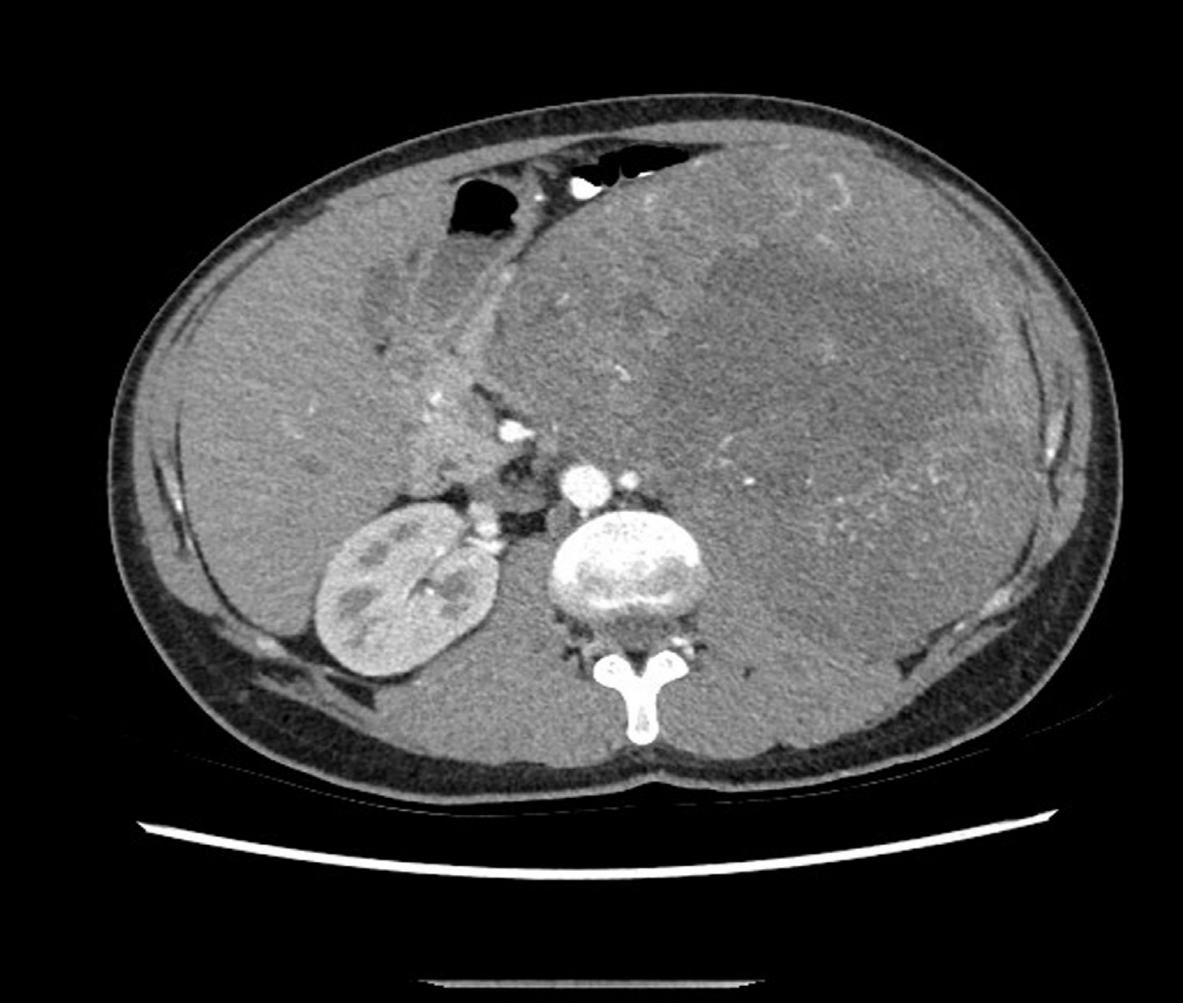
High resolution diagnostic Computed Tomography (HRCT) showed a large isodense mass in the left upper abdomen with areas of hypo-attenuation inside

A 37-year-old man who had a recent history of inguinal hernioraphy and surgery for varicocele referred for follow up. He had no history of previous major medical illness and had an uneventful previous surgery. A high resolution diagnostic Computed Tomography (HRCT) showed a large isodense mass in the left upper abdomen measuring 19 cm in short axis diameter with areas of hypo-attenuation inside (Figure 1). 

The initial blood and biochemistry investigations such as full blood count, renal, liver function tests results and blood tests results for adrenal hormones including Cortisol, Aldosterone, DHEA and Androgenic Steroids, Epinephrine (Adrenaline) and Norepinephrine (Noradrenaline), Thyroid function tests and Calcitonin level are shown in table 1.

Biochemical blood tests results were nearly normal and with a bulky adrenal mass the possibility of adrenal carcinoma was raised and accordingly F-18 FDG PET/CT was requested for detection of possible additional site of involvement. 

The FDG-PET-scan was performed after 6 hours fasting and serum glucose level was 68 mg/dl prior to the scan procedures. The PET scanning was started 60 minutes after intravenous administration of 370 MBq (10 mCi) of F-18 FDG. CT scan was obtained without oral contrast and no IV contrast was given. The non-contrast CT scans was used for attenuation correction and localization. Images were acquired with 85 mAp on a GE discovery MI-DR 64 slice LYSO-crystal PET/CT scanner. Transaxial, coronal and sagittal PET images were reviewed in conjunction with fused noncontrast CT.

**Figure 2.**
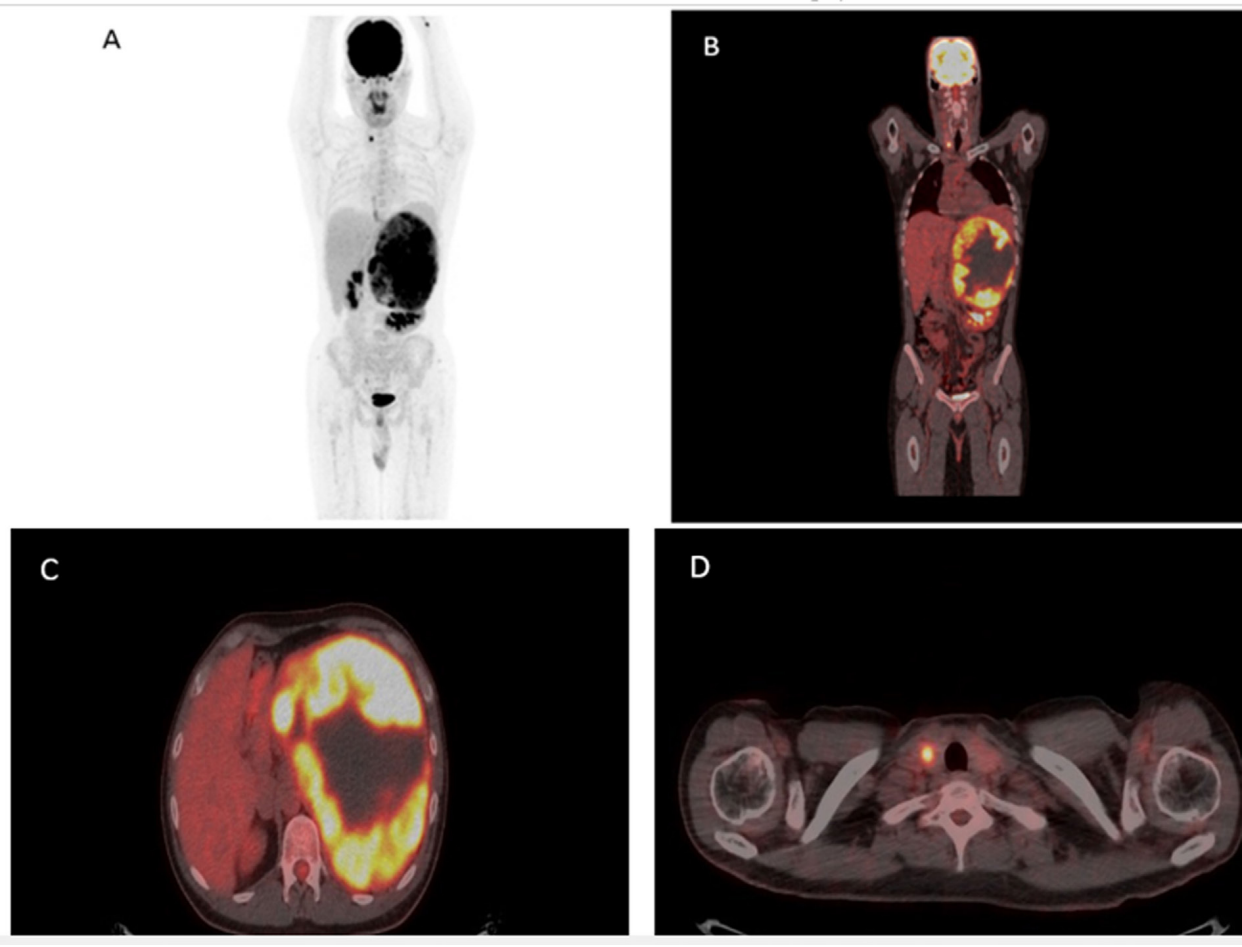
A- D) Maximum Intensity Projection-MIP-PET and PET-CT fused images in coronal and transaxial projections showed left bulky retroperitoneal mass with heterogeneous intense FDG uptake and focal intense FDG uptake in the right thyroid lobe

The maximum intensity projection-MIP-PET and PET-CT fused images in coronal and transaxial projections showed left bulky retroperitoneal mass with heterogeneous intense FDG uptake (SUV_max_ 13.0) and central photopenic areas within the mass (Figure 2 A-C).

In addition, the PET and PET-CT fused images in transaxial projections demonstrated an intense focal FGD uptake within the right thyroid lobe with (SUV_max_ 10.0) (Figure 2 D). There was a low-grade FDG uptake in the distal portion of esophagus with SUV_max_ 3.0 suggestive of gastroesophageal reflux disease. The left kidney was dislocated and was identified in horizontal position immediately below the inferior pole of the suprarenal mass. Otherwise, the scan showed no other abnormal FDG uptake throughout of the body.

**Figure 3:**
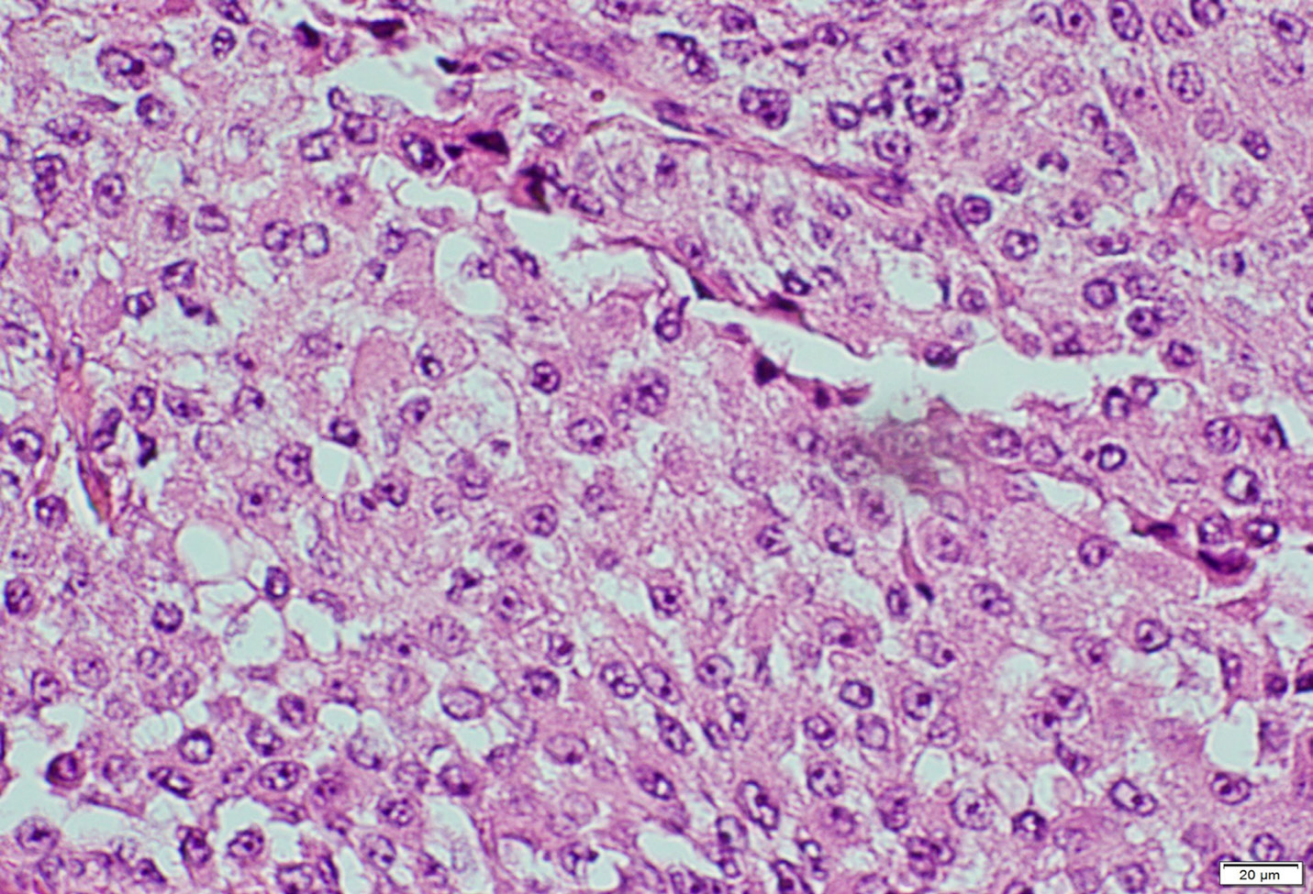
Histopathology slides of adrenal gland oncocytes and classical papillary thyroid carcinoma

**Figure 4:**
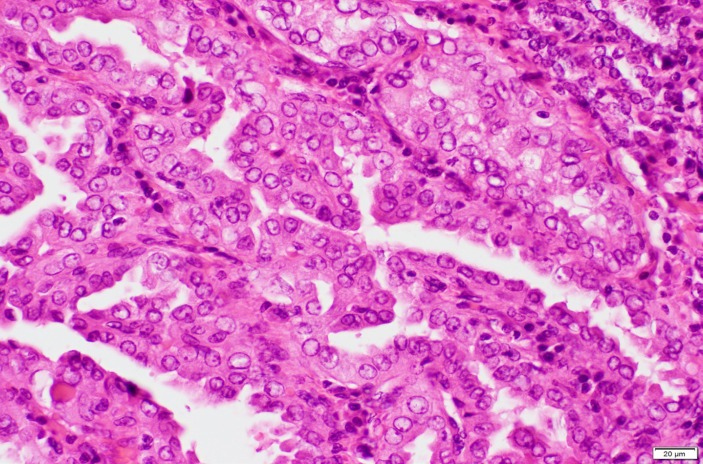
Histopathology slides of adrenal gland oncocytes and classical papillary thyroid carcinoma

He underwent laparotomy and total left adrenalectomy was done and histopathological results revealed adrenocortical carcinoma, oncocytic type (pT2), (Figure 3). Subsequently fine needle aspiration biopsy of the thyroid nodule showed papillary thyroid cancer, and total thyroidectomy was performed. Pathology specimen revealed well-differentiated papillary thyroid cancer, classic type (pT1N0), (Figure 4). 

## Discussion

Double endocrine carcinoma is very rare and has been reported in multiple endocrine neoplasm (MEN) syndromes (5). Simultaneous incidence of Pheochromocytoma and medullary thyroid carcinoma is well known in MEN type 2 syndrome. There are also few reports of papillary thyroid carcinoma associated with Pheochromocytoma in the literature (5-7). A case of aldosterone secreting adrenocortical carcinoma with papillary thyroid carcinoma is also reported (8); furthermore a case of an androgen secreting benign adrenal oncocytoma with concomitant papillary thyroid cancer has been published (9) however, we could not find any report of simultaneous nonfunctioning oncocytic adrenal carcinoma and papillary thyroid carcinoma. 

**Table 1:** Initial blood results of the 37-year-old male before FDG-PET-CT

**Parameter**	**Result**	**Reference range**	**Parameter**	**Result**	**Reference range**
WBC (10^3^/uL)	7.6	3.6 - 11.0	Albumin(g/dl )	4.0	3.4-4.8
RBC (10^3 ^/uL )	4.4	4.50 - 6.0	Total Bilirubin(mg/dl)	0.4	0.1.0
Hematocrit (% )	38.1	40.0 - 52.0	Urea(mg/dL)	21	12-40
Hemoglobin (g/dl)	12.5	13.0 - 18.0	Creatinine(mg/dL)	0.9	0.7-1.2
Platelet (10^3^/uL)	311	150-410	Metanephring (ng/l)	< 50	< 90
ACTH-AM (pg/mL)	< 5.0	<46.0	Normetanephrin (ng/l)	77	< 129
Total Protein(g/dl)	8.0	6.6-8.7	Adrenalin (ng/l)	< 20	up to 84
Dopamine (ng/l)	<20	up to 85	Noradrenalin (ng/l)	353	up to 420
Renin(uIU/mL)	23.0	2.9-30.4	TSH (uIU/mL)	0.63	0.55-4.78
Calcitonin(pg/ml)	10.5	up to 8.4	SHBG (nmol/L)	10.0	9-55
Cortisol- (ug/24hr)	198	6.0-75	Free Androgen Index	114.0	40-150

 Papillary thyroid carcinoma is the most common endocrine malignancy and about 10% of the patients may have distant metastasis mainly to the lungs and bone. Anyhow, adrenal metastasis from papillary thyroid carcinoma is also reported. (10, 11) In our case, pathological examination revealed that it is not a metastasis from thyroid papillary carcinoma (Figure 3).

Oncocytomas are rare tumors, usually found accidentally in different organs particularly in the kidney, thyroid and gastrointestinal tract. They are generally benign with uncertain incidence and potential for malignancy. They may rarely be found in adrenal gland and mostly are non-functioning (1).

FDG is the most common tracer used in positron emission tomography-PET imaging. F-18 FDG uptake is increased in malignant lesions due to high utilization of glucose by malignant cells. Computed tomography-CT is used for evaluation of adrenal lesions and MRI is frequently performed for further characterization. F-18 FDG PET/CT imaging plays a certain and vital role in characterizing malignant from benign adrenal lesions in incidentally detected adrenal tumors in CT or MRI (2). FDG PET/CT showed remarkable outcomes in differentiation of adrenal lesions and furthermore offering the benefit of assessment of primary tumor and also further metastases (2). Yet surgical intervention in terms of adrenalectomy is the only verifiable diagnostic and therapeutic option in case of adrenal oncocytoma (9).

FDG uptake in the adrenal tumor usually compared with background blood pool activity.

Accordingly, all lesions with uptake higher than background were accounted for positive findings; however, some benign adrenal lesions show high uptake, resulting in false positive outcomes (2).

Yun et al, showed that FDG PET/CT had a sensitivity of 100%, specify of 94% and an accuracy of 96% in characterizing adrenal lesions. According to criteria used in this study, if the FDG uptake in the adrenal lesion is less than that of the liver or uptake is markedly higher than liver, it can be interpreted by high confidence. In case the uptake is equal or slightly higher than liver uptake, the study can be read as indeterminate because both malignant and benign adrenal condition can demonstrate this pattern of uptake (12).

In different surveys done by Launay et al., they have exhibited that benign adrenal adenomas had lower standardized uptake value (SUV_max_) than in malignant tumors. 

SUV_max_ in adrenocortical carcinoma ranged from 8.61 to14.15. In addition, 73% of such masses contained areas of necrosis (13).

As related to our patient, we have used two criteria for the evaluation of the non-biopsied adrenal mass as follows: a) the adrenal standardized uptake value which was high and heterogeneous and b) central photopenia within the mass consistent with central necrosis.

Additionally, we observed the obvious incidental finding of a focal intense FDG uptake in the right thyroid lobe corresponding to a small thyroidal nodule in the right thyroid lobe. The incidental finding of focal uptake within the thyroid gland was reported in about 1.5% of FDG PET/CT imaging in a large retrospective series from Italy (14). They found that SUV_max_ of 4.8-7 may be the best cutoff for diagnosis of malignant thyroid nodule and the specificity was increasing in higher SUV_max_ levels (14). 

In another large retrospective study from Turkey the prevalence of thyroid incidentaloma using F-18 FDG PET/CT was reported 5.6% and malignancy risk of incidental thyroid lesions was calculated as 32.7% with proved biopsy or thyroid surgery. According to this group a SUV_max_ above 6 was likely suggestive of malignant findings. However in his study overlap between benign and malignant group was highlighted due to high uptake and accordingly high SUV_max_ in some benign thyroid lesions such as Hurthle cell and thyroiditis. Accordingly incidental FDG uptake outside of target lesions including thyroid, isn’t uncommon in view of physiologic properties of FDG (15).

Moreover incidental findings of thyroid uptake are being explored also with radiolabeled peptide tracer Ga-68-Dotatate. A target group with history of neuroendocrine tumors (NETs) were evaluated for focal and furthermore diffuse thyroid uptake because of difference in biodistribution of Ga-68-Dotate in contrast to F-18 FDG. Accordingly patient with abnormal focal thyroid uptake underwent further assessment and findings revealed thyroid cancer and also benign thyroid tumors or lymphocytic thyroiditis while patients with diffuse Ga-68-Dotate uptake had history of hypothyroidism (16).

In our case with accidental finding of focal high thyroid uptake with SUV_max_ of 10, there was a high potential for a primary thyroid carcinoma. The results were confirmed after total thyroidectomy with histopathology and main histological finding of well-differentiated papillary thyroid cancer (Figure 4).

Other interesting findings in PET/CT images of our patient was FDG uptake in lower portion of the esophagus suggesting gastroesophageal reflux and/or esophagitis that may be the result of pressure effect from large left adrenal mass. 

In conclusion, we are reporting the first case of nonfunctioning oncocytic adrenal carcinoma and papillary thyroid carcinoma in an asymptomatic patient, which is detected by F-18 FDG PET/CT. The adrenal oncocytic adenocarcinomas are rare tumors with variable FDG uptake and mainly managed by surgical removal and furthermore needs regular clinical, biochemical and imaging follow up to rule out local recurrence or distant metastasis. The incidental finding of thyroid lesions in FDG is not rare and use of non FDG tracers resulted in increased rate of thyroid cancer detection in particular in focal thyroid uptake which needs further evaluation to rule out malignancy. 
